# Differential reduction of psychological distress by three different types of meditation-based mental training programs: A randomized clinical trial

**DOI:** 10.1016/j.ijchp.2023.100388

**Published:** 2023-05-14

**Authors:** Christian Liebmann, Annika C. Konrad, Tania Singer, Philipp Kanske

**Affiliations:** a Clinical Psychology and Behavioral Neuroscience, Faculty of Psychology, Technische Universität Dresden, Chemnitzer Straße 46, 01187 Dresden, Germany; b Max Planck Society, Social Neuroscience Lab, Bertha-Benz-Str. 3, 10557 Berlin, Germany; c Max Planck Institute for Human Cognitive and Brain Sciences, Stephanstraße 1a, 04103 Leipzig, Germany

**Keywords:** Psychological distress, Mindfulness-meditation, Mental training, Machine learning

## Abstract

**Objective:**

There is little knowledge about which types of meditation-based training are effective for alleviating which facets of psychological distress. We investigated shared and specific effects of three meditation-based training programs on distress.

**Method:**

332 healthy adults were assigned to a retest control cohort or to one of three 3-month mental training cohorts including: the cultivation of mindfulness-based attention (*Presence*), socio-affective skills such as compassion (*Affect*), or metacognitive skills such as perspective taking (*Perspective*). A battery of 68 self-reported distress measures was collected. Data were analyzed using machine learning methods, identifying the cohort allocation based on distress change scores.

**Results:**

Supporting only specific and not shared alleviation effects, the classifiers identified significantly above chance *Presence* from *Affect* and *Affect* from *Perspective*, but they did not identify the training cohorts from the retest cohorts.

**Conclusions:**

The classifiers revealed stable module-associated distress change profiles, which could help to precisely choose meditation-based interventions to target individuals’ specific distress patterns.

## Introduction

Psychological distress describes a range of symptoms and experiences in a person's internal life that are commonly held to be troubling, confusing, or out of the ordinary (Chaddha et al., 2016). Such distress can have many faces, such as anxiety, depression, bodily complaints, or stress, that, even if it is subclinical, is often sought to be overcome by the individual. Psychological distress or subsyndromal symptoms can evolve into more severe mental disorders. For example, individuals with subclinical depressive symptoms are about four times more likely to meet major depression criteria within two years than those without such symptoms (Cuijpers, 2004). Considering the dimensional nature of psychopathology, in terms of severity and temporal course of symptoms, early treatment of subclinical symptoms seems to be crucial (Besteher et al., 2017). Targeted health programs can prevent psychological distress from progressing into severe mental disorders, reducing future burdens for individuals and society. Prevention programs that are easily accessible, cost-effective, and suitable for diverse distress concerns and cultural backgrounds are essential (Galea et al., 2020).

Recently, meditation-based programs like Mindfulness-Based Stress Reduction (Kabat-Zinn, 1982) and Mindfulness-Based Cognitive Therapy (Segal et al., 2018) have been shown to improve mental health. These programs demonstrate a wide range of benefits for self-reported stress, subclinical (Khoury et al., 2015) and severe symptoms (Goldberg et al., 2018) of various mental health issues, including common disorders like depression and anxiety. Benefits have also been found for people being confronted by adverse lifetime events, including the COVID-19 pandemic (Matiz et al., 2020; Reyes et al., 2020). Overall, meditation-based training has been shown to be equally effective or superior to other established health programs (Goldberg et al., 2018).

There is limited knowledge about the specific effects of different meditation-based mental training types on mental health and psychological distress. Studies exploring differential effects are scarce and indirect. Some research suggests that mindfulness-based and self-compassion-based meditation training may have distinct impacts on mental health. In certain correlational studies, trait self-compassion was a stronger predictor of mental health than trait mindfulness (Baer et al., 2012; Van Dam et al., 2011). In one experimental study, change in self-compassion was a stronger predictor of psychological distress improvement than mindfulness, however, subjects only practiced mindfulness meditation (Galla, 2016).

An exception is the ReSource Project (Singer et al., 2016), a large-scale, 9-month, longitudinal, mental training study that included three different types of mental training and tested differential effects in 332 subjects before and after each of the three 3-month training modules. Thus, the past ReSource Project results have, for example, shown differential effects of three meditation-based training modules, with regard to a) self-reported mindfulness and compassion (Hildebrandt et al., 2017), b) reduction of social stress and the hormonal cortisol level ()(Engert et al., 2017), c) increases in structural brain plasticity (Valk et al., 2017), e) boosting of aspects of prosocial and cooperative behavior ()(Böckler et al., 2018), or f) associated phenomenological reports of meditation-related experiences (Przyrembel & Singer, 2018; Singer & Engert, 2019 for a review). However, so far, no data analyses have focused on testing differential effects of the training regimes on markers of psychological distress such as anxiety, depression, problems with pain, or bodily complaints as assessed through well-known clinically relevant questionnaires.

More specifically, we aimed to use the ReSource Project data set to explore both what was common to the three meditation-based modules (*Presence, Affect* and *Perspective*) as well as the unique effects of each module on training-related changes in markers of psychological distress. Because mental distress can have many facets, all questionnaires, which refer to a clinically relevant distressing trait or prolonged state, should be analyzed. This resulted in a broad spectrum of 68 facets of psychological distress, captured by 16 questionnaires, spanning depressiveness, pain problems, chronic stress, attention and impulsivity problems, physical complaints, and more.

This large number of variables brings conventional study designs and analysis procedures such as ANOVAs and multi-level designs to the methodological and statistical limits of sensitivity (Hindman, 2015). However, machine learning procedures can handle a large number of variables. We thus chose a multi-step machine learning procedure that was designed to classify the respective cohorts based on the induced changes in the 68 psychological distress variables. The procedure was also able to identify those change variables, which were most stable associated with the respective training groups resulting in specific training effect profiles.

We hypothesized that the three training modules would exhibit both shared and unique effect patterns on various psychological distress measures. To examine the first research question regarding shared effects, the machine learning method should predict whether a participant belongs to the retest control group or one of the aggregated training modules based on individual change patterns in distress facets. For the second research question about specific differential effects, the machine learning method should deduce the practiced training module for a participant using these individual change patterns. Additionally, we aimed to explore change variables contributing to training module separation and the magnitude of these training modules' effects on these change variables.

## Methods

### Study design and intervention

**Fig. 1:**
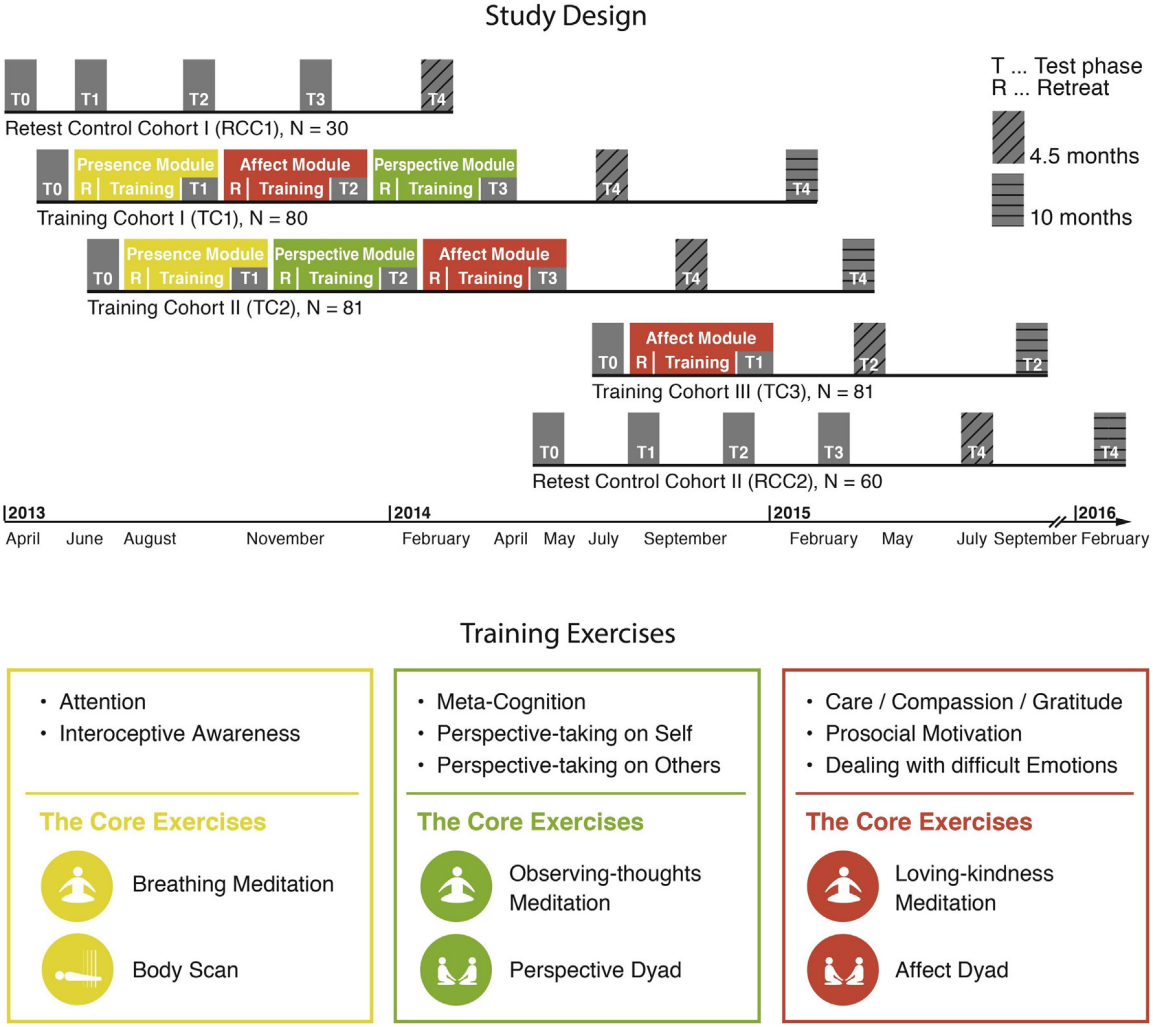
ReSource Project - Conceptual Model and Timeline.

The design of the large-scale, 9-month, mental training study, the *ReSource Project* (for details about the rationale and theoretical backbone see Singer et al., 2016), allowed us to assess differential effects of three 3-month meditation-based training modules (*Presence, Affect, Perspective*) in a conservative and controlled design. Participants were assigned to one of three training cohorts (TC1, TC2, TC3) or a retest control cohort (RCC). The three modules focused on cultivating different kinds of mental capacities, such as a) mindfulness-based attention and interoceptive body awareness (*Presence*), b) loving-kindness, compassion, dealing with difficult emotions, and prosocial motivation (*Affect*), as well as c) meta-cognitive skills and perspective taking on self and others (*Perspective*). The full study design is depicted in Fig. 1.

Each module consisted of two core meditation practices in which participants were required to practice five times a week at home and once a week in two-hour group sessions with professional meditation teachers. These core exercises were: (1) breathing meditation and body scan for *Presence*, (2) loving kindness meditation and the *Affect* Dyad for *Affect*, and (3) observing-thoughts meditation and a *Perspective* Dyad for *Perspective*. A detailed description of the meditation techniques of the three modules can be found in the Supplements.

### Participants

Within two recruitment waves, a total of *N* = 332 healthy participants (197 female; Mean age = 40.74, SD = 9.24; age range = 20-55) participated in the study (see Singer et al., 2016, for a detailed description of the multi-step recruitment and screening procedure and characteristics of the final sample for each cohort). Since this multi-method study involves a large range of outcome variables, the sample size was determined prior to recruitment based on practical considerations and pilot studies and exceeds previously used sample sizes. Participants were assigned to the RCC (*N* =90), TC1 (*N* = 80), TC2 (*N* = 81), or to TC3 (*N* = 81). Assignment was done using a bootstrapping process, which ensured that all cohorts were matched for age, gender, marital status, income, IQ, and several personality trait questionnaires (see Singer et al., 2016). Missing data occurred due to study dropout/exclusion (6%) and technical, health, or scheduling issues at individual assessments (4%). All participants gave informed consent prior to participation and the study was approved by the Research Ethics Committee of the University of Leipzig (number 376/12-ff) and the Research Ethics Committee of the Humboldt University in Berlin (numbers 2013-02, 2013-29, and 2014-10). The study was registered with the Protocol Registration System of ClinicalTrials.gov (Identifier: NCT01833104).

### Self-reported data on psychological distress

For the present manuscript, the clinically relevant questionnaire measures assessed in the ReSource Project were analyzed. In total, 16 out of 49 questionnaires were identified as clinically relevant, covering a broad range of psychological distress symptoms: ADHD Self Report Questionnaire (Rösler et al., 2005), Narcissistic Personality Inventory-40 (Raskin & Terry, 1988), Borderline Personality Questionnaire (Poreh et al., 2006), Machiavellism Scale (Henning & Six, 1977), Mental Health Continuum Short Form (Keyes, 2009), Pain Catastrophizing Scale (Meyer et al., 2008), Satisfaction With Life Scale (Glaesmer et al., 2011), Intolerance Of Uncertainty Scale (Gerlach et al., 2008), Beck Depression Inventory II (Hautzinger et al., 1994), Trier Inventory for Chronic Stress (Schulz et al., 2004), UCLA Loneliness Scale (Russell et al., 1980), Perceived Stress Scale (S. Cohen et al., 1983a), Pittsburgh Sleep Inventory (Buysse et al., 1989), Freiburg Bodily Complaints Inventory (Fahrenberg, 1975), Toronto Alexithymia Scale (Bagby et al., 1994), and State-Trait Anxiety Inventory X1-State (Laux et al., 1981). All the overall scale scores and subfacets were included in the analysis, which resulted in 68 distress variables in total (see Supplements Table S1). For each measure, change scores for each participant in each module were calculated by subtracting individual post- minus pre- module scores (descriptive statistics are provided in the Supplements Table S2 and Table S3).

### Statistical analysis

Data analysis utilizing R software (R Core Team, 2013) comprised a multi-step, machine learning-based approach (see Supplementary Figure S2). The full procedure is detailed in the Supplements. Briefly, four statistical models were repeatedly trained on 68 psychological distress variables' change scores from training data sets, classifying cohort allocations (e.g., Presence vs Affect module) for participants in hold-out test datasets based on individual change scores. A 10-fold cross-validation was performed 10 times to ensure stability.

During two of the four models' cross-validation, a variable filter selection process, based on proposed four-stage approach of Cohen et al. (2020), identified stable effect profile patterns. This process selected variables considered most informative for classifying cohort allocation by at least two of the three algorithms (Random Forest, Bayesian Regression Tree, and Elastic Net). This majority vote ensured methodological independence of the results from the applied algorithms. These preselected variables underwent further filtering for stable predictiveness during a bootstrap-based forward and backward selection process, ensuring the replicability of the discovered effect profiles. This entire four-stage variable selection was executed in 200 cross-validation repetitions. Distress change variables selected in 95% of 200 repetitions were considered replicable and included in subsequent logistic regression analyses. The resulting regression coefficients constituted the first key metric for interpretation. The second significant metric was the mean classification accuracy (balanced accuracy, BAC) of the four classifier models tested against chance level with a nonparametric Wilcoxon Test.

For the first research question on shared effects across three training modules on distress marker changes, classifiers predicted group allocations as training or retest cohorts. Regarding the second question on differential change effects, models first classified participants in *Presence* or *Affect* modules, and then *Affect* or *Perspective* modules. The *Presence* and *Perspective* modules were not compared directly, as the study design did not counterbalance these two modules (due to financial and time constraints).

In line with new data regulations (General Data Protection Regulation, GDPR), we regret that our data cannot be shared publicly because we did not obtain explicit participant agreement for data-sharing with parties outside the Max Planck Institute Leipzig. The present work is based on personal data (age, sex, and medical data) that could be matched to individuals. The data is therefore pseudonymized rather than anonymized and falls under the GDPR. Data are available upon request (contact via corresponding author email address). The code that supports the findings of this study is available from the corresponding author upon request.

## Results

### Shared effects of the three types of meditation-based training

**Fig. 2:**
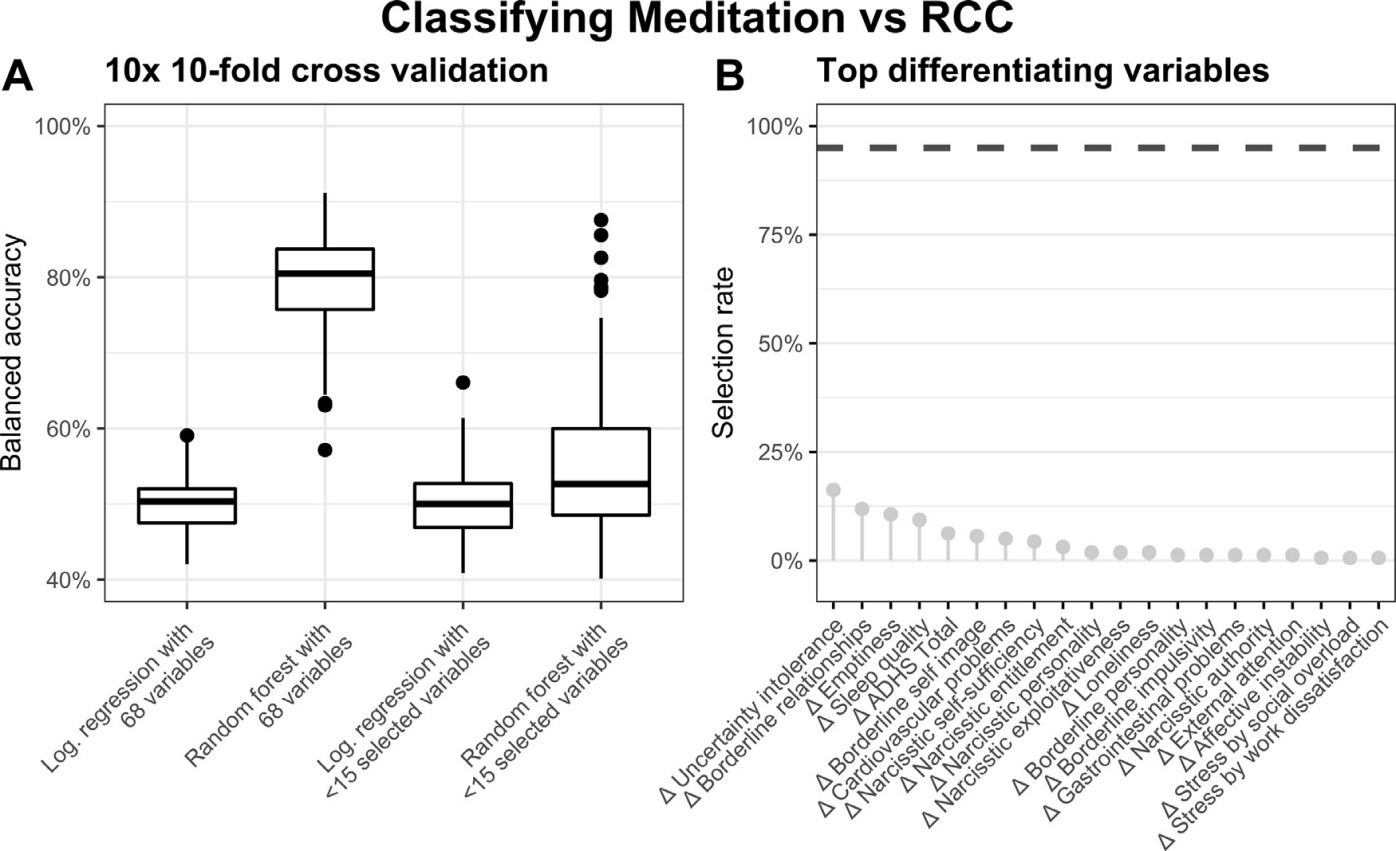
Shared effects of the three aggregated meditation-based training modules. (A) Boxplots show mean balanced accuracies of four classifiers predicting allocation of aggregated meditation modules vs. retest control cohort based on the individuals’ distress change scores. (B) Lollipop diagram shows highest selection rates resulting from the predictor selection procedure during cross-validations.

For the first question regarding shared effects across the three training modules on psychological distress markers ("shared effects"), classifiers predicted cohort allocation to aggregated training or retest groups. The balanced accuracies (BAC) of logistic regressions (Median = 50%, all *p*s > 0.7) were at chance level (see Fig. 2A). Although the unfiltered random forest's BACs interquartile range was not above chance, the median was significantly greater than 0.5 (Median = 53%, IQR = [49%, 60%], *p* = 0.0002). In contrast, the unfiltered random forest considering all 68 distress change variables achieved a BAC interquartile range significantly above chance level (Median = 80%, IQR = [76%, 84%], *p* < 0.0001). However, no distress variables survived the filtering variable selection process in at least 95% of cases (Fig. 2B). This result pattern indicates that the three mental training regimes—focusing on a) mindfulness-based attention, b) socio-emotional, and c) socio-cognitive skill training—lacked shared common main effects on psychological distress within our 68-distress variable set.

### Specific effects of the three training modules

#### Differential effects of the presence and affect module

**Fig. 3:**
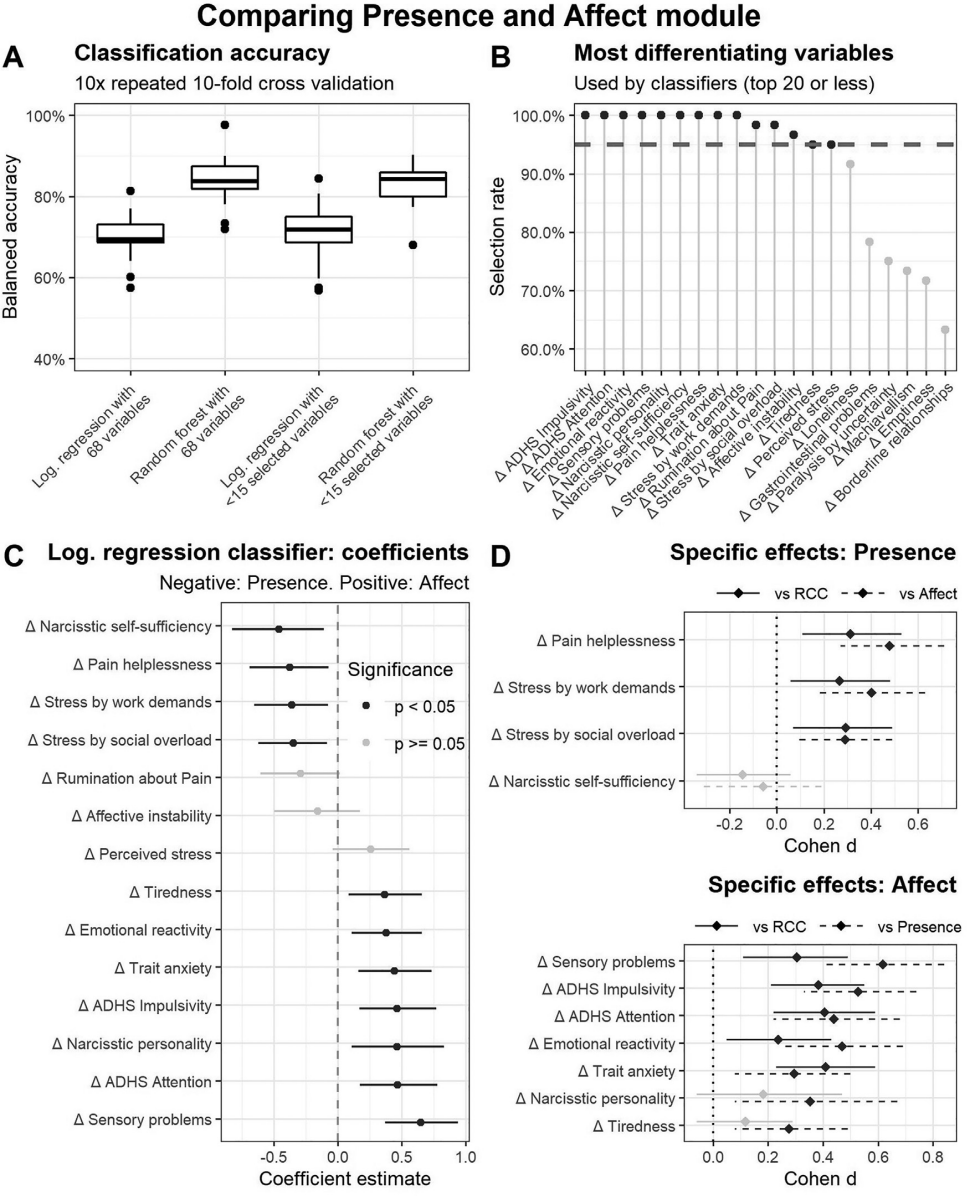
Analysis of differential effects of Presence and Affect module. (A) Boxplots show mean balanced accuracies of four classifiers predicting allocation of Presence vs. Affect module based on the individuals’ distress change scores. (B) Lollipop diagram shows highest selection rates resulting from predictor selection procedure during cross-validations. (C) Coefficients and standard errors of a logistic regression are displayed by using 14 variables that reached the level of a 95% selection rate (see B) as predictors. Negative coefficients indicate the practice of Presence module, positive coefficients indicate the practice of the Affect module. (D) Effect sizes and *t*-tests of the candidate variables for specific effects were calculated by comparisons to the respective other module and the retest cohort.

For the second question of specific change effects, in the first step, the classifiers predicted the cohort allocation to the *Presence* cohort or the *Affect* cohort. All average classifiers’ BACs were significantly above chance level (Medians between = 70 and 84%, IQRs min = 66%, IQRs max = 89%, all *p*s < 0.0001). The BACs varied mainly between the type of statistical model of the classifier, but not between applying the variable selection process or not (see Fig. 3A). Fourteen variables were selected in 95% of the cases of the variable selection during the cross-validations (Fig. 3B). Four of these 14 variables showed significant coefficients with negative signs in an exemplary logistic regression, namely narcissistic self-sufficiency, pain helplessness, stress by work demands, and stress by social overload. This indicates that people with positive changes in these distress measures completed the *Presence* module (Fig. 3C). Seven of the 14 variables showed significant positive coefficients, indicating that the person with improvements in these facets practiced the *Affect* module, which were sensory problems, ADHD-like attention problems, narcissistic personality, ADHD-like impulsivity, trait anxiety, emotional reactivity, and tiredness. For both sets of variables, posthoc effect sizes and *t*-tests in comparison to the other training module and the retest group were calculated. If the size of the effect was significantly greater than 0, in comparison to the other module and the retest group, we interpreted these distress change variables as belonging to the specific effect profile of the respective module. This was the case with the *Presence* module for reducing helplessness in relation to pain, stress by work demands, and stress by social overload (Fig. 3D). Here, the effect strengths varied between 0.23 and 0.25, and thus in a small range.

The significant specific effects of the *Affect* module were sensory problems, ADHD-like attention and impulsivity problems, emotional reactivity, and trait anxiety. The effects varied between 0.2 and 0.4 and are therefore in the small range.

#### Differential effects of the Affect and the Perspective module

**Fig. 4:**
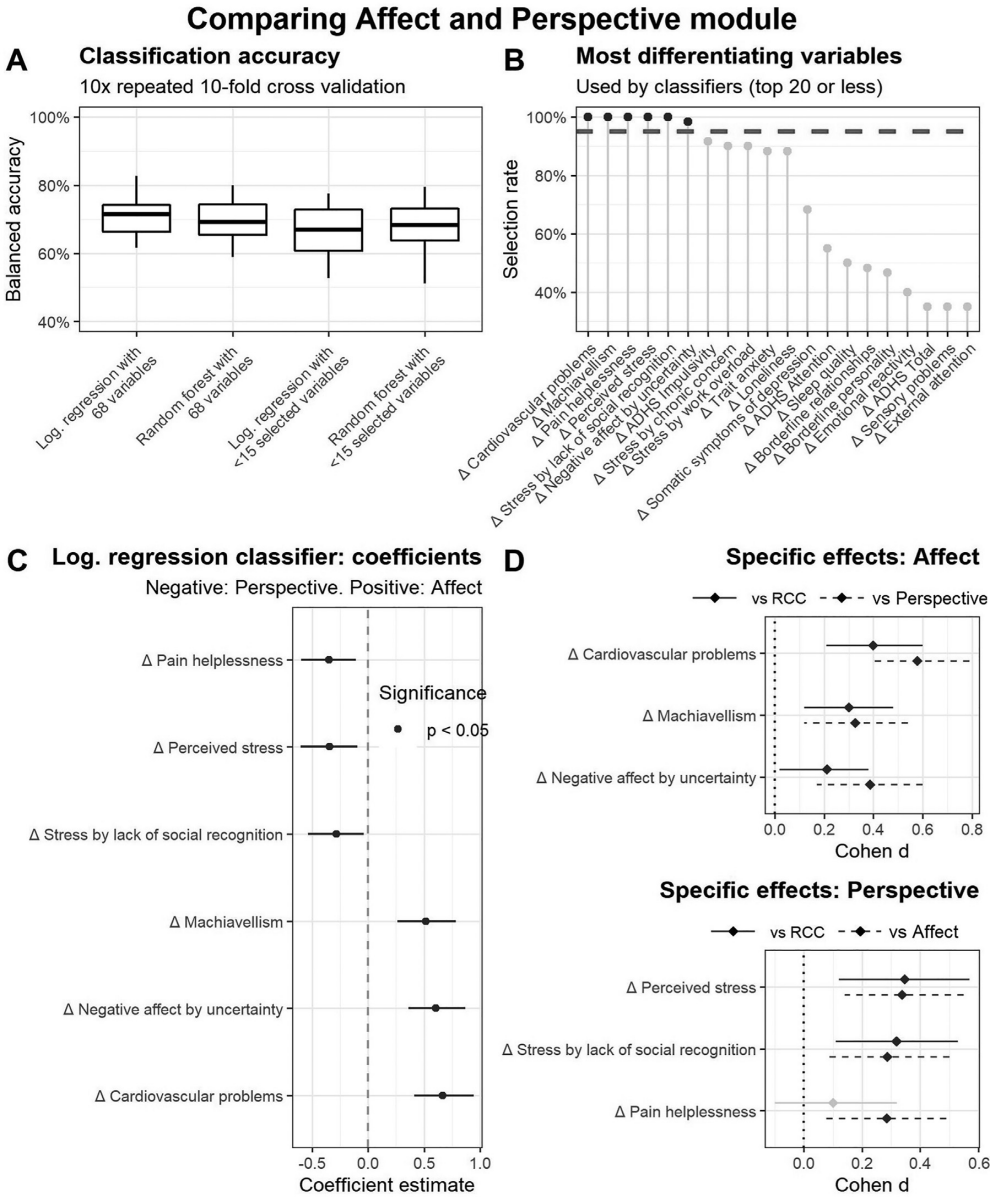
Analysis of differential effects of Affect and Perspective module. (A) Boxplots show mean balanced accuracies of four classifiers predicting allocation of Affect vs. Presence module based on the individuals’ distress change scores. (B) Lollipop diagram shows highest selection rates resulting from predictor selection procedure during cross-validations. (C) Coefficients and standard errors of a logistic regression are displayed by using six variables that reached the level of a 95% selection rate (see B) as predictors. Negative coefficients indicate the practice of Perspective module, positive coefficients indicate practice the of Affect module. (D) Effect sizes and *t*-tests of the candidate variables for specific effects were calculated by comparisons to the respective other module and the retest cohort. Significant effects are displayed in black instead of grey.

For the question of differential alleviating effects, in the second step, the classifiers predicted the cohort allocation to the *Affect* or the *Perspective* group. The details of the following results are depicted in Fig. 4. The average classifiers’ BAC was significantly above chance level (Median = 66%, IQR = [61%, 72%], *p* < 0.0001). All classifiers’ BACs were significantly above chance level and hardly varied between classifier type, and application or omission of the filtering variable selection (Medians between 64% and 69%, IQRs min = 60%, IQRs max = 74%, all *p*s < 0.0001, see Figure 4A). Six variables were selected during cross-validation in 95% of the cases of filtering classifiers (see Fig. 4B). Three of these six variables showed significant coefficients with negative signs in an exemplary logistic regression, which means that individuals with positive change scores in these distress facets indicate having completed the *Perspective* module, namely pain helplessness, perceived stress, and stress by lack of social recognition (see Fig. 4C). The other three of the six selected variables showed significant coefficients greater than 0, indicating that individuals had practiced the *Affect* module, namely cardiovascular problems, negative affect by uncertainty, and machiavellism. For these two differential sets of variables, post-hoc effect sizes and *t*-tests in comparison to the other module and the retest group were calculated (see Fig. 4D). Here, effect sizes of the *Perspective* module were significant in both comparisons for the variables of perceived stress and stress by a lack of social recognition with effect sizes between 0.34 and 0.31, equaling a small range.

**Fig. 5:**
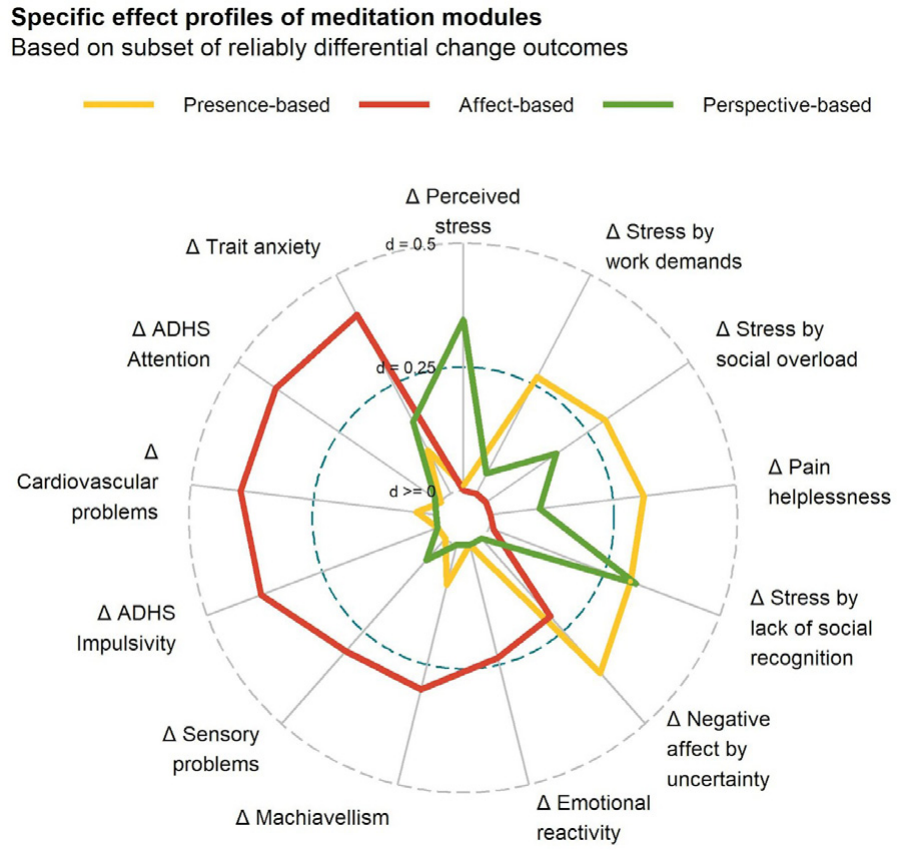
Radar chart shows specific effect profiles of meditation modules. The distance to the center point constitutes the effect size Cohen's *d* of the respective module in comparison to the retest control cohort.

The significant specific effects of the *Affect* module, with effect sizes greater than 0, were improvements in cardiovascular problems, reduction in machiavellism, and negative affect by uncertainty. The effect strengths were between 0.2 and 0.4 and were thus in the small range. An overview of the specific effects and their sizes can be found in Fig. 5.

## Discussion

This study examined the shared and distinct effects of three mental training regimes on a broad range of psychological distress markers related to stress, anxiety, depression, and other aspects assessed through self-report questionnaires. The first contemplative training module focused on mindfulness-based attention (Presence module) through breathing meditation and body scan. The second module targeted socio-affective capacities like compassion (Affect module) through daily loving-kindness practices and empathy-building partner exercises (Affect Dyad). The third module encompassed training of metacognitive capacities (Perspective module) with observing thoughts meditation and a 10-min partner activity emphasizing perspective-taking abilities.

Utilizing machine learning approaches, four classifier models predicted participants' module type based on computed distress change scores from 16 different questionnaires and their subscales, resulting in 68 measures. To examine the shared effects of all three training modules, the machine learning procedure predicted whether a participant belonged to the retest control group or one of the aggregated training modules based on individual distress facet changes. The second research question aimed to identify differential effects of the training modules, with machine learning methods predicting a participant's practiced module.

Our analysis revealed that the type of practice was crucial in reducing various psychological distress aspects (see also Singer & Engert, 2019). The investigated mental training modules exhibited no common main effects on psychological distress but rather displayed distinct differential change profiles. Our results indicated that in case of 13 distress measures, there were differentiating, specific effect profiles of the modules. Specific training-related changes in different psychological markers occurred after participants completed certain training modules. Consequently, there were no shared effects allowing classifiers to differentiate the aggregated training modules from a retest control group, with accuracies at chance level. In contrast, classifiers distinguished between Presence and Affect modules with 77% average accuracy, and between Affect and Perspective with 67% accuracy. Thus, the results demonstrate that the three meditation-based training modules tested within the ReSource Project (Singer et al., 2016) were effective in their unique ways to reduce different self-reported mental health risk aspects and psychological distress in healthy adults. Similarly, the findings also highlight the ineffectiveness of certain mental training types on specific psychological distress aspects, emphasizing the importance of accurately indicating particular mental training procedures. Nevertheless, some limitations must be noted, when interpreting the results. First, the generalizability of the results across different ethnicities and races cannot be guaranteed, because we lack data from the participants on their ethnicity. Second, even though the overall sample size is large, the subgroups are rather small with *N* = 80 in the TC1 and *N* = 90 in the RCC.

However, when looking more closely at the specific effects of the training modules, the current findings provide a basis for informing others about potential indications for specific mental training practices. Our results suggest that pain-related helplessness (subscale of the Pain Catastrophizing Scale), self-reported stress from work demands, social overload, lack of social recognition (subscales of the Trier Inventory for Chronic Stress, TICS), and problems related to negative affect due to uncertainty (Intolerance Of Uncertainty Scale) can be most effectively mitigated by training mindfulness-based attention to present body sensations (breath meditation, body scan) or other sensory objects in the present moment (meditation on sounds, visual objects, or taste), as practiced in the ReSource Presence module. The Perspective module led to effects on distress, but with fewer markers and smaller effect sizes. Both modules incorporated focused attention meditations traditionally included in the Mindfulness-Based Stress Reduction (MBSR) program (Kabat-Zinn, 1982). Our findings align with prior knowledge about MBSR's effects, with meta-analytic evidence suggesting that MBSR helps healthy individuals cope with pain (Veehof et al., 2016) and reduces self-reported stress (Khoury et al., 2015). Another ReSource Project study, which incorporated biological stress markers (Engert et al., 2017), revealed that practicing the Presence module resulted in a higher covariance between subjective stress response and the stress hormone cortisol following exposure to an acute social stressor. These findings indicate that the Presence module may have assisted participants in better identifying everyday situations that triggered individual psychoendocrine stress responses, potentially promoting more effective coping with daily stress.

In the present study, the Presence module had a stronger impact on certain distress markers, such as pain-related helplessness, stress due to work demands, and stress caused by social overload, but did not differ regarding effects on general subjective stress from the Affect module. Comparing these findings with those of Roca et al. (2021), both studies found meditation-based interventions to be effective in reducing stress. However, the Presence module, which focuses on attention-related exercises, affected specific stress markers more than general stress perception. In contrast, results from Roca et al. (2021) suggested that Mindfulness-Based Stress Reduction (MBSR) affected overall subjective stress. The Presence module includes a subset of MBSR exercises, but omits components such as open monitoring meditations, psychoeducation, and communication. These differences indicate that targeted attention and body-awareness exercises may better address specific stress markers, while comprehensive mindfulness practices and group discussions may effectively manage overall stress perceptions.

The discovery of improved uncertainty management aligns with the Buddhist origins of the MBSR program, which aims to cultivate equanimity in an ever-changing world (Eberth et al., 2019). Research on MBSR's impact on coping with uncertainty has primarily focused on physical medical diagnoses such as breast cancer (Janusek et al., 2019) and infertility (Mousavi et al., 2020). Our findings imply that strengthening uncertainty coping skills is possible even in the absence of current health issues, offering promising avenues for enhancing resilience during future health challenges.

In contrast, the compassion-based socio-emotional Affect module exhibited the most extensive effects on distress-related measures, including trait anxiety, ADHD-like impulsiveness, attention issues, cardiovascular complaints, emotional reactivity, sensory sensitivity, and machiavellian behavior. The socio-emotional and compassion-based training module demonstrated the most comprehensive and robust effects on psychological distress aspects. This finding aligns with prior ReSource Project results, which revealed the Affect module's unique, training-related impact on compassion improvement, related brain networks, altruistic cooperation, helping behaviors, emotion regulation capacities, and self-compassion. The increased influence of the Affect module on attention and impulsivity issues was somewhat unexpected, as mindfulness-based attention practices are typically associated with heightened attention. A systematic review linked mindfulness meditation practices with enhancements in selective, executive, and sustained attention abilities (Chiesa et al., 2011). Thus, it may be surprising that the Affect module demonstrated a more substantial impact on attention than the mindfulness-related Presence module. However, additional ReSource Project evidence shows equal improvement in attention performance after three months of Affect and Presence training (Trautwein et al., 2020), suggesting that compassion-based practices are equally effective in enhancing attention.

Unexpectedly, the Affect module reduced self-reported cardiovascular sensations complaints (e.g., chest pain, heaviness, or palpitations during exertion) and sensory problems (color, odor, or taste sensitivity). We utilized the Freiburg Bodily Complaints Inventory to measure individual tendencies to complain and experience bodily discomfort, correlating with neuroticism and emotional liability (Fahrenberg, 1975). Our results suggest that the Affect module decreases the inclination to complain and worry about cardiovascular and sensory sensations. This is in line with previous research showing that compassion training has positive impact on heart rate variability (HRV; Kirby et al., 2017; Petrocchi & Cheli, 2019; Steffen et al., 2021), which, in turn, seems to be associated with feelings of safeness and social connectedness. Thus, the reduction in cardiovascular-related concerns may be due not only to actual changes in HRV, but also to pleasant micro-phenomenological experiences reported during kindness-related meditation around the heart region (Przyrembel & Singer, 2018), potentially replacing worries about ambiguous body sensations.

## Conclusion

Subclinical psychological distress, capable of escalating into severe mental disorders, imposes significant costs on individuals and society. Our findings indicate that meditation-based training can improve multiple dimensions of psychological distress, including stress, anxiety, pain-related helplessness, bodily complaints, attention and impulsivity issues, and intolerance of uncertainty.

Furthermore, in our research, machine learning algorithms revealed the distinct effects of three types of training regimes (attention-based, socio-emotional, and socio-cognitive). Alleviation of stress and pain-related problems was linked to mindfulness-based attention practices, while socio-cognitive training also impacted some stress-related issues. The compassion-based socio-affective training module demonstrated the most extensive range of training-related effects, encompassing concentration and impulsivity issues, emotional reactivity, anxiety, antisocial behaviors, sensory sensitivity, and cardiovascular concerns.

In the future, meditation-based prevention effectiveness could increase by considering the specific training effects detected for individuals targeting particular mental health challenges.

## Declaration of Competing Interest

The authors declare that the research was conducted in the absence of any commercial or financial relationships that could be construed as a potential conflict of interest.
